# A power supply module for autonomous portable electronics: ultralow-frequency MEMS electrostatic kinetic energy harvester with a comb structure reducing air damping

**DOI:** 10.1038/s41378-018-0025-2

**Published:** 2018-09-24

**Authors:** Yingxian Lu, Frédéric Marty, Dimitri Galayko, Jean-Marc Laheurte, Philippe Basset

**Affiliations:** 1 Université Paris-Est, ESYCOM (EA2552), CNAM, ESIEE Paris and UPEMLV, Noisy-le-Grand, 93162 France; 2 Sorbonne Université, LIP6 lab, CIAN group, Jussieu campus, Paris, 75005 France; 3 Université Paris-Est, ESYCOM (EA2552), CNAM, ESIEE Paris and UPEMLV, Marne-la-Vallée, 77420 France

## Abstract

A MEMS electrostatic kinetic energy harvester (e-KEH) of about 1 cm^2^, working at ultralow frequency (1–20 Hz), without any supported additional mass on its mobile electrode, and working even without a vacuum environment is reported. The prototype is especially suitable for environments with abundant low frequency motions such as wearable electronics. The proposed e-KEH consists of a capacitor with a finger-teeth interdigited comb structure. This greatly reduces the air damping effect, and thus the capacitance variation remains important regardless of the presence of air. With the new design, the energy transduced per cycle of excitation is no less than 33 times higher than the classic design within 10–40 Hz/2 *g*_peak_, while is 85 times higher at 15 Hz/2 *g*_peak_. An enclosed miniature ball combined with non-linear stoppers enables the oscillation of the movable electrode through impact-based frequency up-conversion mechanism, which is also improved by the low air damping. Thanks to this new design, a higher efficiency than the classic gap-closing comb structure is obtained, as a larger range of working frequency (1–180 Hz) in air. A maximum energy conversion of 450 nJ/cycle is obtained with a bias voltage of 45 V and an acceleration of 11 Hz, 3 *g*_peak_. Working with a diode AC-DC rectifier, the proposed KEH is able to support up to 3 RFID communications within 16 s while operated at 11 Hz, 3 *g*_peak_.

## MEMS: harvesting kinetic energy

A MEMS-based electrostatic kinetic energy harvester (e-KEH) offers high energy conversion via a capacitor with an integrated finger-like structure. Energy generation is vital for self-powered electronic devices. However, small devices for kinetic energy harvesting are most effective at high-frequencies, whereas environmental kinetic energy is distributed over a large range of frequencies, especially at the low end. A team led by Philippe Basset at Université Paris-Est in France now reports an e-KEH device that is highly effective in the low frequency range. In addition, the key finger-like capacitor structure – enabled by a new fabrication process – is less affected by air dampening, enabling it to function effectively even in a non-vacuum environment. Such a power supply module design could be advantageous in self-powered portable systems.

## Introduction

A growing number of portable and wearable electronics results in an increasing demand of sustainable power supplies. The demands for these power supplies include high output power, small size and weight, and well adapted to the environmental excitations. There is a growing interest in making use of the environmental energy such as kinetic energy, radiation, and thermal energy^1^. Among them, the environmental kinetic energy is distributed in a wide frequency range, especially in low frequencies^2^. And for wearable electronics or implantable devices, ultra-low frequency vibrations or motions (mostly < 20 Hz) are the most abundant sources available for energy conversion. However, the available electrical power of kinetic energy harvesters (KEHs) is proportional to the excitation frequency, the mass of the movable part and the mass displacement, all of which being limited quantities^3^.

The techniques to improve the efficiency of a KEH depend on its energy transduction module, which can be piezoelectric^4^, electromagnetic^5^, or electrostatic^6-9^. The electrostatic KEH (e-KEH) is advantageous regarding microscale designs. It can be modeled as a DC-biased variable capacitor, transducing energy when the mass movement opposes to the electrostatic force inside the capacitor. Its efficiency is determined by the evolution of the charge stored in the transducer and the voltage across it (i.e., the QV cycle) during the capacitance variation^10,11^. There are two ways to improve the efficiency: to create more QV cycles in each period of mechanical excitation (i.e., frequency-up conversion) through impacts^12,13^ or through bistable structures^13,14^ and to convert more energy in each QV cycle by increasing the ratio of capacitance variation *η* = *C*_max_/*C*_min_^9,15,16^, by providing a higher bias voltage^7^, or achieving a preferable geometry of the QV cycle^17^. Here *C*_max_ and *C*_min_ are the maximum and minimum capacitance of the transducer.

The capacitance of MEMS e-KEHs are typically realized in the form of comb-shaped electrodes. In 2006, we proposed an e-KEH with an In-Plane Overlap-Plate (IPOP) structured, where the overlapping area between the comb electrodes changes with the in-plane motion of the movable electrode^18^. The energy transduction was 1.4 nJ per mechanical cycle at 0.25 *g*_rms_ 250 Hz. *η* for this prototype was limited by the invariable gap between electrodes (*η* = 2) and the low pull-in voltage (12 V). A second generation of e-KEH based on mono-layer silicon structure with gap-closing interdigital comb electrodes was reported^19^. The converted energy was 15 nJ/cycle at 1 *g*_rms_ 150 Hz and a maximum bias voltage of 30 V. A third-generation e-KEH sharing the same dimensions as the previous generation with elastic stoppers^20^ and a mini-ball^21^ was proposed. The pull-in voltage was higher (46 V) because the impact of the ball counteracts the electrostatic force. A frequency-up conversion behavior was observed in low frequencies (below 50 Hz)^21^. Thus, the efficiency was improved, especially at low frequency: The energy converted in each cycle of excitation was 55 nJ at 2 *g*_rms_ 11 Hz and 30 nJ at 5 Hz. However, the air damping force on the gap-closing combs was significant, limiting the frequency-up effect at low frequencies and the capacitance variation at low acceleration, and thus the output power.

In this work, we propose e-KEHs with a new comb structure that greatly reduces the squeeze film air damping, so that the costly vacuum package is not required for good energy conversion performance. A new process based on SOI wafers achieves a higher silicon etching aspect-ratio and a higher electrode surface area for a same device area. The converted energy per cycle of excitation with this new comb geometry is more than one order of magnitude higher than that of the previous gap-closing prototypes in air.

**Fig. 1:**
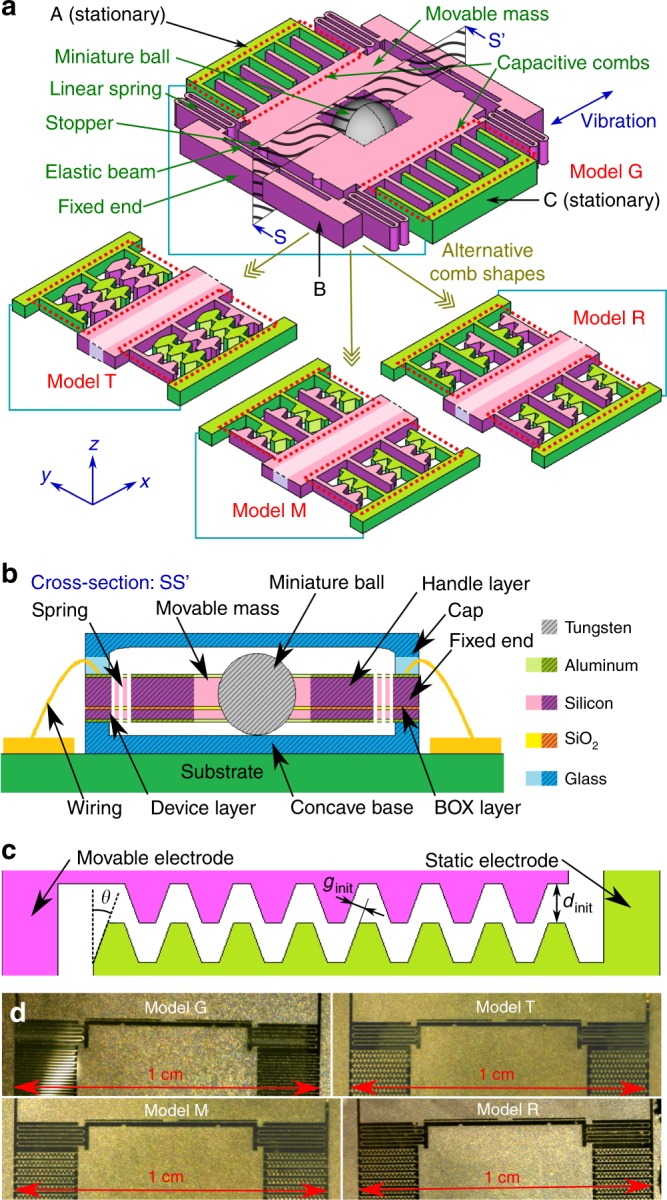
**a** Simplified 3D schematic of the kernel structure of the e-KEH; **b** View of the full device from section SS’ in **a**; **c** The key design parameter of the hierarchical comb structure: side angle *θ* of the teeth. **d** Microscopic photographs of the KEHs

The e-KEHs as shown in Fig. 1 are developed from the prototype reported in ref. ^21^. A simplified schematic of the devices is shown in Fig. 1a, and a cross-section view of it is shown in Fig. 1b. The kernel structure of the e-KEH is developed from an SOI wafer, consisting of three parts: A, B, and C, each of which embodies an electrode. Parts A and C are fixed, and part B is located between A and C, containing a movable mass. The corners of the movable mass are connected to fixed ends through linear springs. The movable mass vibrates along *x* axis, and its displacement is limited by elastic stoppers on the two fixed ends. In the center of the movable mass, there is a cavity holding a miniature ball. The entire structure is packaged by a concave base and a cap, so that the mini-ball is maintained within the cavity. When the e-KEH is driven at any frequency, the resonant oscillation of the movable mass can be started whenever there is an impact from the ball. Thanks to the frequency-up conversion brought by the ball, the vibration can be triggered even when the excitation frequency is far lower than the natural frequency. The adjacent sides of A/B and B/C are interdigital combs, forming variable capacitors *C*_AB_ and *C*_BC_ between the electrodes. A DC bias voltage is applied on the electrode in B. While the electrodes in A and C are electrically connected with each other. Under this configuration, the vibration of the movable mass is transformed to electric energy when the capacitor is electrically charged.

Instead of applying the classic gap-closing structure in the interdigital combs, we introduce hierarchical comb structures. Here the expression “hierarchical comb structure” refers to a 2-level comb structure of fingers and teeth where rows of hexahedral teeth are distributed equidistantly along the length of comb fingers. The classic gap-closing comb structure is applied in the first prototype to work as a reference. Three new designs with hierarchical structure are proposed for comparison regarding the implementation of the hierarchical structure. *Model T* is the design where both sides of each comb are modified to hierarchical structures. In the other two configurations, each comb has the teeth array only on one side. In one configuration, the direction of teeth along *X* axis in capacitor *C*_AB_ is the same as that in capacitor *C*_BC_. The prototype is mirror-symmetric and is named as *Model M* accordingly. For the other configuration, the directions of teeth on the movable electrode in capacitors *C*_AB_ and *C*_BC_ are opposite to each other, and the prototype is called *Model R* due to its rotational symmetry. Detailed design parameters of the designs can be found in the supplementary material.

## Materials and methods

### Fabrication of e-KEHs from SOI wafers

The fabrication of the proposed e-KEHs is based on SOI wafers where the thicknesses of the handle layer, the buried oxide (BOX), and the device layer are 380 μm, 2 μm, and 100 μm, respectively. An aluminum thin film is deposited by sputtering on both sides of the wafer, and patterned to serve as an etching mask layer. The two silicon layers are then fully etched through Deep Reactive Ion Etching. The exposed BOX layer is etched by vapor hydrogen fluoride (HF) to release the movable structure. A glass wafer is processed into a concave structure below the mobile parts through sand blasting, and anodically bonded to the device layer of the SOI wafer. The device is then deposited with a layer of Parylene C through LPCVD process (working as insulator and electret material). The movable electrode is corona charged. A miniature ball is placed in the central cavity of the movable mass, and a glass cap pre-etched by hydrofluoric acid solution is glued on top of the prototype. Eventually, the device is glued to a PCB substrate. The microscopic photographs of the 4 prototypes (before capping) are shown in Fig. 1d. More information about the fabrication process can be found in the supplementary material.

### Optimization of comb design

A simplified top view of the hierarchical comb structure is shown in Fig. 1c. The initial gap between the fixed and movable electrodes along *x* axis in all the 4 designs are identical (*d*_init_) for all the four prototypes, and the length of teeth are identical. In addition, the four prototypes all share the same design for the springs and the elastic stoppers (initial gap on the stoppers are 66 μm), while the size of the capacitive modules are all identical. The capacitance variations of each model are calculated regarding the displacement of the movable electrode (*x*). The ratio of capacitance variation *η* = *C*_max_/*C*_min_ is affected majorly by the design parameter *θ*, where *C*_max_ and *C*_min_ are maximum and minimum capacitance, respectively. The capacitance of the four models are calculated by finite element analysis.

The *C*_min_ of the 4 models are roughly equivalent to each other (*C*_min_ ≈ 25 pF). The influences of *θ* on *C*_max_ are calculated considering the constrains for layout geometry as stated above. *θ* is the decisive factor of the capacitance variation (*θ*_opt_ = 25.5°, see supplementary material). The initial gap between the teeth sides (*ɡ*_init_) are constantly 30 μm when 0° < *θ* < *θ*_opt_ and it increases with the increasing *θ* when *θ*_opt_ < *θ*< 90°, while the variation of the gap between teeth sides always increases with the increase of *θ*. As a result, the minimum gap between teeth sides decreases with an increasing *θ* from 0° to *θ*_opt_, leading to an increasing maximum device capacitance. In addition, the bottom width of each tooth increases with *θ*, thus the total number of teeth that can be distributed on each comb is reduced, leading to a smaller total capacitance. Considering the large slope of *C*_max_ against *θ* when *θ* < *θ*_opt_, we apply *θ* = 30° in the proposed designs, slightly larger than *θ*_opt_.

The major constrains of the fabrication process for the dimensions in the mask are brought by the deep reactive ion etching process on the handle layer. To ensure the reliability of the structure and the successful release of the moving structure, the widths of both remaining structures and the etched part should be no less than 30 μm. The capacitance variation vs. the mass displacement of the prototypes with the optimal teeth angle (*θ*) can be found in the supplementary material.

### Experimental setups for the KEH characterization

**Fig. 2:**
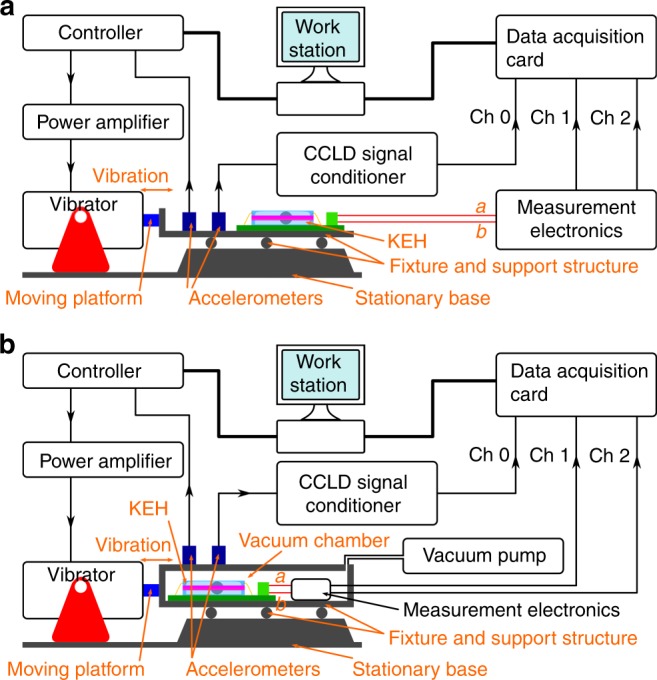
Schematic of experimental setups for the KEH characterization in air **a** and in vacuum **b**

For the electromechanical characterization of the KEH, the schematic of the experimental setup is shown in Fig. 2a, b. The prototype is installed to a fixture which is connected to the moving platform of a shaker, together with measurement electronics and two accelerometers (Type 4507 B 004 from Brüel & Kjær). The vibrator is model V406 from LDS Test and Measurement, and is controlled by a PC through a feed-back signal provided by one of the accelerometers. For the measurements in air (Fig. 2a), the fixture is a horizontal base plate, on which the prototype, the measurement electronics and the accelerometer are fixed. For vacuum measurement (Fig. 2b), the KEH and the measurement electronics (including followers) are installed inside a vacuum chamber so as to minimize the parasitic capacitance; while the accelerometers are installed on the exterior of the chamber. The air pressure within the chamber is maintained to 10^−3^ mbar through a vacuum pump (Model D-35614 from Pfeiffer Vacuum) during vacuum tests. Different types of pre-defined acceleration series are used to excite the vibrator, including sinusoidal signals with either single frequency or frequency sweeps, or acceleration series recorded from human motions during running or walking.

The capacitance measurement is performed on the four KEH devices both in air and in vacuum before corona charging, using the dynamic measuring method described in ref.^22^. The KEHs are excited by single-frequency sinusoidal series of acceleration (with the amplitude of 2 *g*_peak_, and the optimal frequency for each model: 135 Hz for *Model G*, 100 Hz for *Model R*, 140 Hz for *Models M* & *T*). To ensure a periodic capacitance variation, the devices are operated without the mini-ball. Each prototype is loaded with a resistor of 15 kΩ (*R*_load_), and excited by a sinusoidal carrier signal of 75 kHz, 0.5 V_peak_. The signals on the two electrodes of the KEH are recorded, and the dynamic capacitance variation is calculated according to the phase difference between the two signals. Firstly, the capacitances of the device installed on the fixtures in air and in vacuum at stationary state are obtained through dynamic measurement method, respectively. The capacitance of the stand-alone stationary device is also measured by a U1732C LCR meter from Agilent. The difference between these two measurements gives the total parasitic capacitance of the fixture and the measurement electronics *C*_par_. This parasitic capacitance is removed from the capacitance evolution curves measured with dynamic method before plotting.

For AC power measurement, pre-defined acceleration series with constant amplitudes (0.5 *g*_peak_, 1.0 *g*_peak_, and 2.0 *g*_peak_) and sweeping frequencies (either sweeping up or sweeping down within the range between 10 Hz and 600 Hz) are applied on the KEH by the vibrator. The sweeping rate is 1 Hz/s. The models chosen for measurement are the ones offering the maximum capacitance variation ratio either in air or in vacuum (i.e., Models G and R). The prototypes are characterized in both with and without the mini-ball, in air and in vacuum, respectively. The prototype is biased by a DC voltage (varying from 20 V up to 45 V), and loaded with an optimal resistor of 6.6 MΩ. The voltage on the resistive load is read by the data acquisition card, and the average output power corresponding to each frequency is calculated accordingly and recorded by the LabVIEW program.

In AC/DC power conversion experiment, the KEHs are biased with varied DC voltage (from 5 V to 65 V). Single-frequency sinusoidal acceleration with constant amplitude (10 Hz, 2 *g*_peak_) is applied to the vibrator. The output signal of the prototype is rectified by half-wave, and the energy is stored with a reservoir capacitor *C*_res_ = 1 μF. The voltage evolution with time is monitored by the data acquisition card, and recorded by LabVIEW program, and the power is calculated accordingly.

During the experiment of data transmission, the KEH is excited either by a vibrator driven by sinusoidal signals or by the hand shaking motions. The energy from the prototype is rectified and stored in a reservoir capacitor *C*_res_ = 1 μF, and the voltage across it (*V*_res_) is monitored by the data acquisition card. The energy in *C*_res_ is released to a data transmission module through a manually controlled mechanical switch, as long as the voltage reaches the maximum allowable voltage, and the power supply is suspended when *V*_res_ drops below the minimum allowable voltage^23^. The data communication module implements an RFID chip EM4324 provided by EM Microelectronic, the working voltage of which is 1.1 V to 3.3 V. The RFID chip communicates with a remote reader under the framework of EPC Gen-2 Class-1 standard at the frequency of 868 MHz. The tag reading is performed periodically by an Impinj reader from a distance above the maximum distance of passive reading (2.5 m). The emission power of the reader is 1 mW, and the time slot between two tag readings is 38 ms.

The measurement electronics can be found in the supplementary materials, including the circuits for capacitance measurement, AC power measurement, AC/DC power conversion and data transmission, respectively. The model of the amplifiers (U_1_ and U_2_) is OPA445 from Texas Instruments, while the diodes are low-leakage models PAD5 from Vishay. Up to two channels of electrical signals in the measurement circuits and the output signal of the accelerometer are recorded by a data-acquisition card USB-6366 from National Instruments, and processed by LabVIEW programs.

## Results

### Capacitance variation

**Fig. 3:**
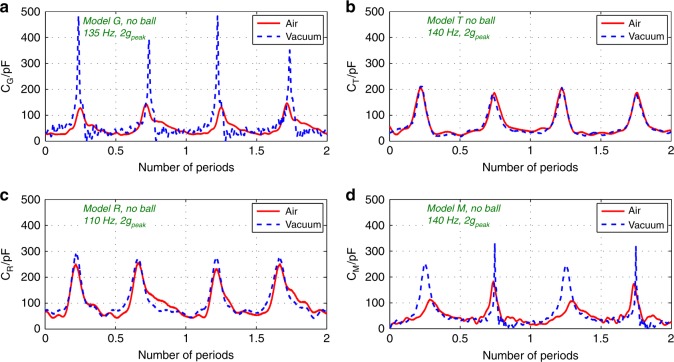
Capacitance variation of the Models G (**a**), T (**b**), R (**c**), and M (**d**) (without DC bias voltage) without the mini-ball in air and in vacuum

The capacitance variations of the four models without the ball are tested, in air and in vacuum, under the acceleration of 2 *g*_peak_ and optimal frequencies, with the best performance in air and in vacuum achieved by *Models R* and *G*, respectively. The transient curves are shown in Fig. 3. The parasitic capacitance (28 pF) is removed from the measured values. It is observed that the minimum capacitances of the four models are *C*_min_ = 25 pF as predicted in the theoretical calculations. In contrast, *C*_max_ (the average peak capacitances) of the four models varies with the comb shapes and the maximum displacement of the movable mass, which is affected by the air damping effect. In vacuum, the air damping effect can be neglected, so that the capacitance variation ratios *η* = *C*_max_/*C*_min_ of the four models agree with the theoretical calculations: *Model G* reaches the highest ratio (*η* = 17.6, *C*_max_ = 440 pF), corresponding to the maximum displacement of 69 μm. In air (with standard pressure), the air damping force obstructs the motion of the movable mass, reducing its maximum displacement. Thus, the ratio *η* for each model in air is lower than that in vacuum.

The influence of air damping in *Model G* is the most significant among the four models: its peak capacitance in air is 130 pF, less than one third of that in vacuum, corresponding to a displacement of only 64 μm. In addition, the time duration of each pulse of capacitance in air is much larger than that in vacuum, due to the hyperbolic C(x) function in the gap-closing comb structure, which also results from the small mass displacement caused by large damping force of air. The falling edge of the capacitance pulse is less steep than rising edge, indicating a reduced velocity during the travel from the maximum displacement back to the balance point, which is also an evidence of the air damping effect: The direction of the air damping force, unlike the spring forces, is always opposite to the direction of the movable electrode’s velocity, obstructing the capacitance changes. The air damping force majorly comes from the smaller air gap^24^. As a result, the absolute value of the movable electrode’s acceleration is larger in the approaching stage (rising edge) than that in the receding stage (falling edge). This leads to the time duration of the rising edge of the capacitance curve smaller than the falling edge, i.e., the rising slop is steeper in the timeline. Consequently, the duration of the falling edge is larger than the rising one.

The capacitance variation of *Model T* in air is very similar to that in vacuum (*η* = 8, *C*_max_ = 200 pF), indicating that the hierarchical comb shape is advantageous in reducing the air damping effect. The approaching motion of the hierarchical combs is a combination between sliding and gap-closing motion, leading to a reduced squeeze film air damping effect, and consequently a reduced damping force. The new comb shape reduces the relative velocity between electrode facets. However, the *C*_max_ of *Model T* is limited because of a limited number of combs for the same area.

The design achieving the maximum capacitance ratio in air is *Model R* (*η* = 10.8, *C*_max_ = 270 pF), thanks to the collective effect of a larger number of combs and a reduced air damping force from the hierarchical combs. The relative difference between its peak capacitances in air and in vacuum is less than 10%.

Although the hierarchical comb structure is applied in *Model M* as in *Models R* & *T*, the air damping force is still significant: its average *C*_max_ in air (120 pF) is only 52% of that in vacuum (*C*_max_ = 230 pF). In this prototype, all the planar sides of the combs approach each other simultaneously, leading to a strong air damping force like in *Model G* but 50% of the time. A performance summary of the four models is provided in the supplementary materials.

### Energy conversion with frequency sweeps

**Fig. 4:**
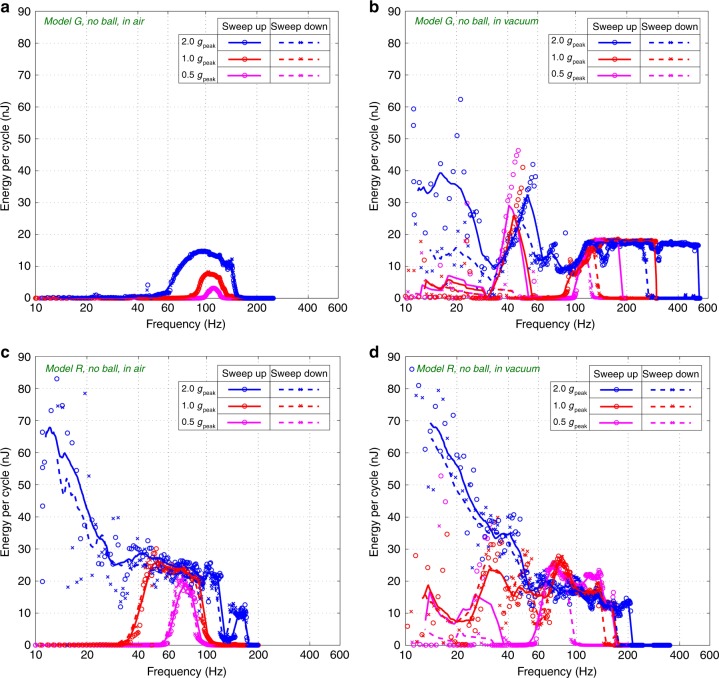
Frequency sweeps with resistive load: Energy per cycle of the *Models G* (**a**, **b**) and *R* (**c**, **d**) (biased at 20 V) without the mini-ball in air (**a**, **c**) and in vacuum (**b**, **d**) under varied acceleration: 0.5, 1.0, and 2.0 *g*_peak_

**Fig. 5:**
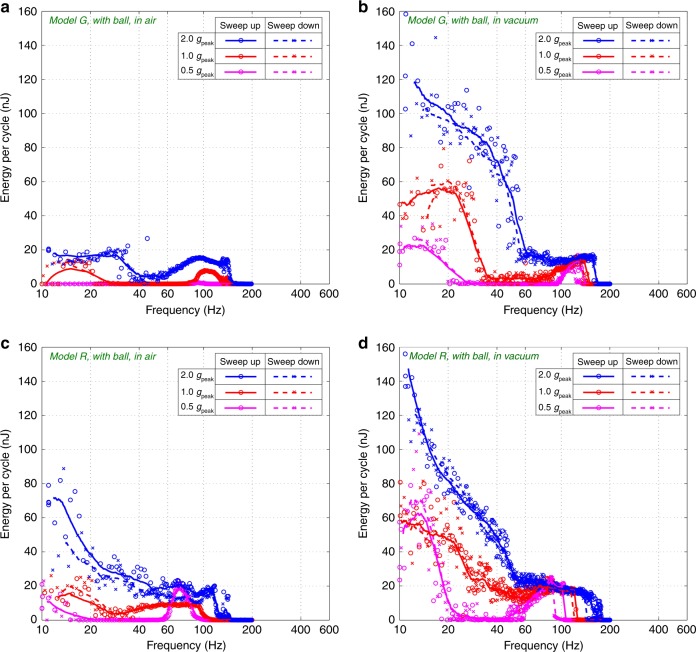
Frequency sweeps with resistive load: Energy per cycle of the *Models G* (**a**, **b**) and *R* (**c**, **d**) (biased at 20 V) with the mini-ball in air (**a**, **c**) and in vacuum (**b**, **d**) under varied acceleration: 0.5, 1.0, and 2.0 *g*_peak_

Figure 4 shows the energy converted by the models *R* and *G* without the mini-ball through frequency sweeps. The e-KEH are biased at 20 V and submitted to accelerations with varied amplitudes (0.5 *g*_peak_, 1 *g*_peak_, and 2 *g*_peak_), in air and in vacuum, respectively. Figure 5 shows the measurements when the prototypes work with the ball, sharing all the remaining conditions as for Fig. 4. The comparison of performances without the ball show the influence of the new comb shape to the air damping effect, while with the mini-ball it demonstrates the collective effect of the new comb design and the impact with the ball. The parasitic capacitance brought by the measurement electronics (*C*_par_ = 28 pF) cannot be excluded simply by calculation as for the dynamic capacitance measurements, so the results are underestimated. The energy per cycle shown in Figs. 4–5 is obtained by dividing the output power by the excitation frequency. The discrete data points (circles and crosses) are the data obtained directly from experiments, and the lines gives the average power of adjacent frequencies.

It is observed from Fig. 4 that the bandwidth of *Model G* is relatively small in air, and the energy conversion drops drastically when the frequency drops below certain thresholds (60 Hz for 2.0 *g*_peak_, 85 Hz for 1.0 *g*_peak_, 100 Hz for 0.5 *g*_peak_). The maximum energy conversion is only 14.7 nJ/cycle at 95 Hz, 2 *g*_peak_. This is due to the high air damping that limits the displacement of the mobile electrode. For this reason, almost no hysteresis is present, however spring-softening effect due to the electromechanical coupling can clearly be observed. In comparison, the range of its working frequency is greatly expanded in vacuum and the energy conversion is improved throughout the whole working frequency range. This is particularly true at low frequency (below 60 Hz): At 20 Hz and 2 *g*_peak_, the energy conversion in vacuum (20 nJ) is about 50 times of that in air (0.4 nJ). This gives an evidence about the considerable air damping effect in *Model G* at low frequencies. Also, the frequency hysteresis is limited, because the electrostatic force is small, majorly due to the large air damping force, which obstructs the gap between electrodes from reducing (the minimum gap of Model G in air is 3 µm according to the analytical calculations).

In contrast, *Model R* has a large range of working frequency, even in air. An increase of energy conversion with the decrease of frequency (frequency-up conversion behavior)^25^ can be easily observed in the entire frequency range at 2 *g*_peak_, especially between 10 Hz and 40 Hz, both in air and in vacuum. This behavior takes place only when the air damping is low enough to allow high impacts on the elastic stoppers. The energy conversions with 2 *g*_peak_ in air and in vacuum are similar, on the contrary that with *Model G*, indicating again a clear reduced air damping effect. The maximum energy per cycle for *Model R* in air is ~66 nJ (at 12 Hz, 2 *g*_peak_), only 14% lower than that in vacuum and about 4.5 times of that for *Model G* in air.

From Fig. 5 it is observed that the insertion of the mini-ball introduces a significant additional frequency-up conversion behavior at low frequency (below 60 Hz), both in air and in vacuum. The energy conversion of *Model G* at 20 Hz and 2 *g*_peak_ is increased by 45 times, from 0.4 nJ/cycle without the ball to 18 nJ/cycle with the ball. In contrast, the power improvement of *Model R* at low frequency and 2 *g*_peak_ is less significant, because the model already has a frequency up-conversion behavior due to the impact with the flexible stoppers. However, with the insertion of the ball, the frequency up-conversion occurs at lower accelerations. The energy conversion of *Model R* in air reaches 12 nJ/cycle at 20 Hz, 1.0 *g*_peak_ with the ball (about 100 times of the one without the ball).

On the other hand, the improvement of energy conversion due to the ball is negligible in the frequency range above 60 Hz: in air, the optimal energy conversion achieved by *Model G* (15.2 nJ/cycle) is only 3% higher than without the ball. As for *Model R*, the maximum power achieved with the ball is even lower than without the ball. This is because of the interruption of vibrations brought by the impacts of the ball. In addition, the frequency hysteresis of *Model G* in vacuum is mostly eliminated by inserting the ball (Fig. 5b), this hysteresis being negligible in air. Similar phenomenon is observed from the curves of *Model R* in vacuum (Figs. 4d and 5d). The cause for this is that the impacts from the ball can easily interrupt the unstable vibrations in the hysteresis region.

**Fig. 6:**
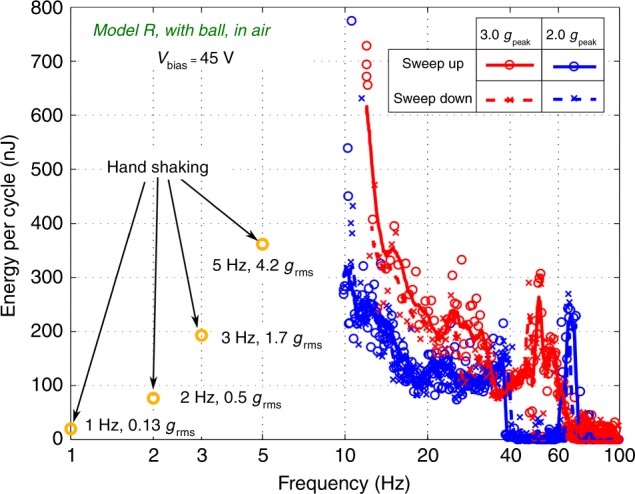
Frequency sweeps with resistive load: Energy conversion of *Model R* in air with frequency sweeps under the accelerations of 2 *g*_peak_ and 3 *g*_peak_, with the maximum allowed bias voltage (45 V)

To explore maximum energy conversion of the prototype, the bias voltage is increased to the highest (45 V). The vibration of the combs is interrupted by pull-in when the bias is further increased. The acceleration is also pushed all the way up to the maximum (3 *g*_peak_). The energy conversion performances of the device under these conditions are shown in Fig. 6. We observe that the energy conversion rate is increased by more than 4 times compared to the performance with 20 V bias, but the range of working frequency is reduced by ~60% (10–68 Hz). A maximum power of 13.2 μW is achieved at 50 Hz, 3 *g*_peak_. Energy drop due to unstable oscillations is observed in the frequency range of 37–64 Hz with 2 *g*_peak_ acceleration, and 63–100 Hz for 3 *g*_peak_. The highest effectiveness^3^ of 54% is achieved at the frequency of 10 Hz with 2 *g*_peak_ acceleration (0.33 μJ/cycle), while for the acceleration of 3 *g*_peak_ is 50% achieved at 12 Hz (0.45 μJ/cycle). The energy conversion with hand shaking motions below 10 Hz are also marked in the figure. 0.36 μJ/cycle is obtained with 5 Hz, 4.2 *g*_rms_ shaking motion. Besides the drop of acceleration, the cause for the energy decrease below 10 Hz is also related to the interruption of the mass oscillation caused by impacts between the movable electrode and the ball. The current prototype is optimized for oscillations around 10 Hz. To further reduce the optimal working frequency, the cavity length should be enlarged.

### AC/DC transduction

**Fig. 7:**
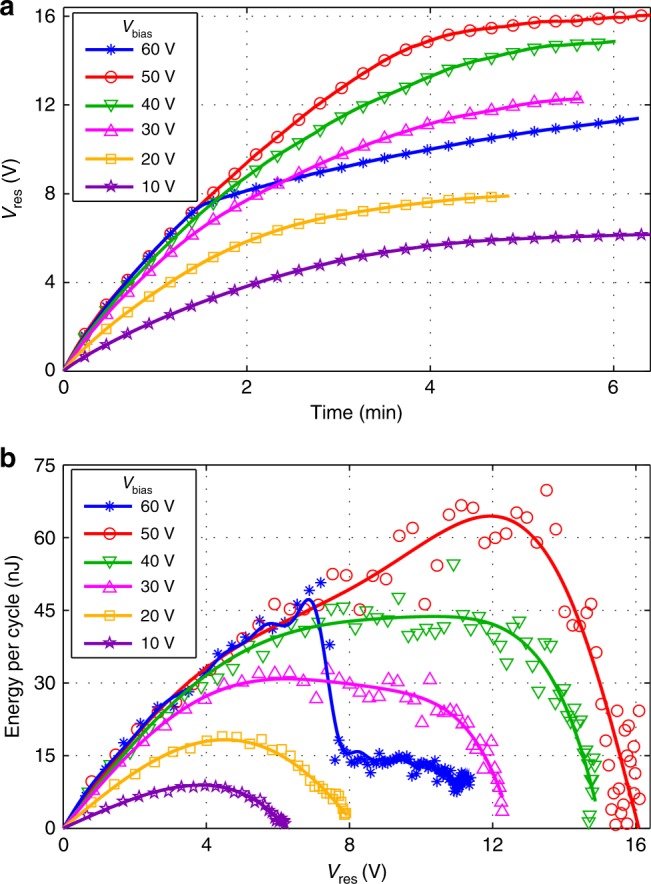
AC/DC rectifying: **a** Evolution of *V*_res_ and **b** average energy per cycle vs. *V*_res_ with *Model R* biased at varied voltage (10–60 V) working with half-wave rectifier (2 *g*_*peak*_, 10 Hz)

Figure 7 shows the results of AC/DC transduction experiments where *Model R* is excited by a sinusoidal acceleration (2 *g*_*peak*_, 10 Hz) in air, working with a half-wave rectifier. The influence of *V*_bias_ varying from 10 V to 60 V is investigated. The transient evolutions of *V*_res_ are shown in Fig. 7a, while the relation between *V*_res_ and the average energy conversion during each cycle of excitation is shown in Fig. 7b.

It is observed that the optimal energy conversion is reached with *V*_bias_ of 50 V and *V*_res_ of 12 V. With these optimal conditions, the converted energy reaches 64.4 nJ/cycle. In each charging curve, the evolution of *V*_res_ firstly experiences a linear growth with time, before slowing down until a maximum energy conversion is reached. Then the voltage growth gradually approaches saturation, and the energy drops with further increase of *V*_res_. This saturation comes from the half-wave diode rectifier, as demonstrated in reference 25. With the increase of *V*_bias_, the initial slope of accumulated energy per cycle vs. voltage increases, but the increment of this slope is unobtrusive when *V*_bias_ exceeds 30 V, with a value of 8 nJ/V. The saturation voltage and the maximum power increase with increased *V*_bias_ below 50 V. With a *V*_bias_ higher than 50 V, the energy conversion is constantly interrupted by the pull-in status of the KEH, so that the average energy conversion is less efficient.

### Data transmission

**Fig. 8:**
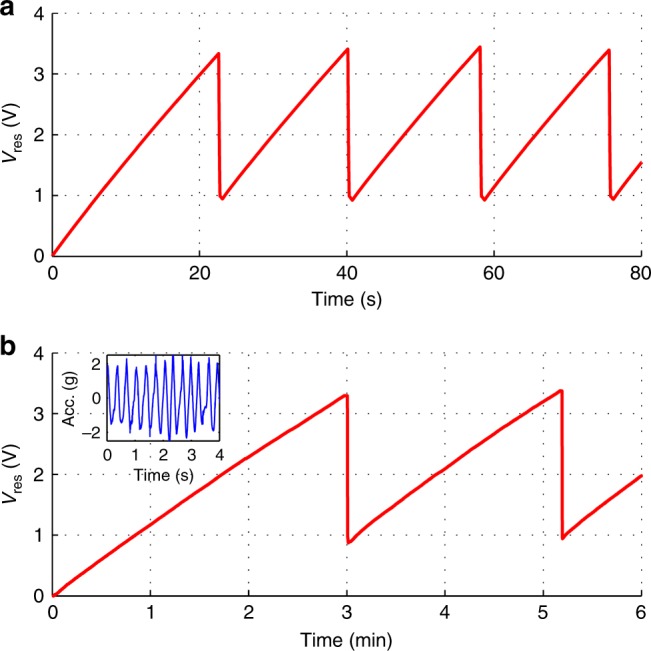
Data transmission: *V*_res_ evolution (energy conservation / release) during data transmission experiment. *Model R* biased at 50 V is excited by **a** a sinusoidal acceleration of 11 Hz, 3 *g*_peak_, **b** a series of acceleration with shaking motion of hand at ~3 Hz, with the average peak value of 2 *g*, the inset shows the acceleration recorded from the hand shaking motion

The evolution of *V*_res_ during the data transmission experiments is shown in Fig. 8, where the *Model R* biased at 50 V works as the power supply. Fig. 8a shows the charging/discharge of *C*_res_ with the KEH excited by a sinusoidal acceleration of 11 Hz, 3 *g*_peak_. The initial charging from 0 V to 3.3 V takes 22.4 s, during which 5.4 μJ is accumulated, corresponding to the average energy conversion of 22 nJ/cycle. The energy consumption occurs only when the mechanical switch is turned on (connected), the RFID tag is read by the remote reader three times in a row, after which *V*_res_ drops from 3.3 V to 1 V (the minimum power supply voltage of the RFID chip), and the RFID tag is unavailable to the reader. During each of the following charging processes, *V*_res_ rises from 1 V to 3.3 V, and the increment of energy stored in *C*_res_ is 4.9 μJ during 16 s. The average energy of 28 nJ is accumulated during each period of the acceleration. Then the mechanical switch is turned off (disconnected), and the accumulation of energy restarts. Since *V*_res_ varies between 1 V and 3.3 V, the half-wave rectifier is working far from the optimal condition (*V*_res_ = 12 V, learnt from Fig. 7b), the energy conversion of the KEH is much lower than the value under condition (64.4 nJ/cycle).

Figure 8b shows the capacitance charging/discharge with the KEH excited by the acceleration of gentle hand shaking motions at a rate of 180 beats per minute, as shown in the inset. The acceleration is featured with repetitive pulses of random waveforms, the average peak acceleration is about 2 g. The initial charging takes 3 min, corresponding to the average power of 30 nW (10 nJ/cycle). Each following chargings takes 2.2 min, corresponding to the average power of 37 nW (12.4 nJ/cycle). The *V*_res_ evolution of the system during a data transmission experiment when the KEH is excited by a random hand-shaking movement can be found in the supplementary materials. These results give us a view of the KEH performance under a practical situation in wearable electronics.

## Discussion

### Dynamic capacitance measurement

In the experiment of capacitance measurement, the accuracy of the results is determined by the accuracy of phase difference between the signals on the two electrodes of the KEH. The calculation is based on the assumption that the capacitance variation is negligible within one period of the input AC signal. The accuracy drops drastically if the capacitance change fast. Moreover, a given resistor *R*_meas_ corresponds to a “target” capacitance, and the error of measurement grows when *C*_var_ moves away from the “target”. In the experiments, we choose *R*_meas_ that makes the “target” capacitance to be right equivalent to the average of *C*_max_ and *C*_min_, so as to minimize the error. However, the results for *Model G* in vacuum shown in Fig. 3a still suffer of inaccuracy: the variation of capacitance is fast when the movable mass is close to the maximum displacement, while the range of capacitance variation is high. The capacitance drops to nearly zero right after each peak, which is not possible according to the device geometry. In order to get a higher accuracy, a higher frequency should be applied to the sampling signal, and the resistor should be adaptive to the capacitance variation.

### Frequency-up conversion

In the proposed KEHs, the frequency-up conversion is initiated by impacts of the movable electrodes: either with the elastic stoppers or with the mini-ball. When the air damping is low, the movable electrode can easily reach the elastic beams to trigger the frequency-up conversion. In this case, the oscillation of the movable electrode is more likely to be interrupted than enhanced by the impacts with the mini-ball. So, the combination of the two frequency-up conversion structures does not improve the power further. The frequency-up conversion can be optimized in two aspects: The length of the cavity can be adjusted, so that the time interval between two adjacent impacts with the mini-ball is synchronized with the oscillation of the movable electrode. Moreover, we can adjust the ratio between the mass of the mini-ball and that of the movable electrode (in particular, increase this ratio for this specific design), so that the oscillation of the movable electrode is less likely to be stopped by its impact with the ball. Thus, the power of the KEHs can be boosted by both frequency-up conversion structures simultaneously with less conflict, so that we can expect a better performance from the KEHs, especially at low frequency (below 50 Hz).

### Air damping effect

The air damping of the hierarchical comb is drastically reduced in contrast with the classical gap-closing prototype. The reason for this is the relative motion between the surfaces of the two electrodes. In gap-closing interdigital combs, the velocity of the movable electrode is perpendicular to the approaching comb surfaces, so the squeeze film air damping model should be applied^24^. In the prototypes with hierarchical combs, the motion of the teeth facets is the combination of gap-closing and sliding motions. Thus, the squeeze-film air damping model is no longer applicable, and the damping force is much smaller. A complete model of the prototypes including the air damping should be developed to facilitate further optimization.

### Conditioning circuit

In the AC/DC transduction experiments, the half-wave diode bridge rectifiers are used because they achieve a higher energy conversion rate than full-wave diode rectifiers. This comes from the fact that at large *C*_max_/*C*_min_, the area of the QV cycle is larger for a half wave rectifier than for a full wave rectifier^16^, while in addition, the voltage drop of diodes in a full wave rectifier is larger than half-wave rectifier.

The optimal performance of energy conversion of the KEH biased at *V*_bias_ = 50 V is reached when the voltage across the reservoir capacitor reaches 12 V. In the data transmission experiment, the DC working point of the KEH is far from the optimal condition: *V*_res_ is directly applied to the following electronics, always no higher than 3.3 V. Therefore the device is less efficient. In order to improve the performance of the KEH in DC power supply applications, the voltage on *V*_res_ should be adjusted to the optimal working point. Additional electronics for DC/DC voltage conversion is needed as interface.

Moreover, an automatic switch^26^ is needed to control the release and conservation of energy, to realize the manual operation of the mechanical switch as in the data transmission experiment. However, both the DC/DC voltage conversion interface and the automatic switch will bring additional power consumption. It is challenging to reduce the leakage current of the switch and the power consumption of the DC/DC converter, considering the high optimal reservoir voltage.

## Conclusions

We have introduced three new models of MEMS-based low frequency electrostatic kinetic energy harvesters (*models T*, *R*, & *M*), containing a hierarchical comb geometry that drastically reduce the air damping force. The design of the comb shape is optimized regarding the devices’ capacitance variation. The prototypes share a unified structure with a movable electrode connected to fixed ends through linear springs, holding a mini-ball in a cavity at the center, and implementing elastic stoppers. The KEHs are based on SOI wafers and a newly developed fabrication process that offers a fabrication resolution higher than that of the previously reported work^26^.

The capacitance variation of the three models with the new comb structure is compared to a prototype with classic gap-closing interdigital combs (*Model G*) both in air and in vacuum and without the mini-ball, so that the prototype achieving the best performance is identified. The minimum capacitances of the four models are approximately equal, so the largest maximum capacitance leads to the highest energy conversion. *Model T* has a smaller *C*_max_ because it has the least number of comb fingers. The performance of *Model G* is optimal in vacuum as predicted in the theoretical calculation, while *Model R* is the most efficient prototype in air, thanks to the greatly reduced air damping force. The AC power of *Models G* and *R* in air and in vacuum is measured with frequency sweeps, both with and without the mini-ball. Frequency-up conversion behavior is observed, brought by impacts either on elastic stoppers or with the mini-ball. The optimum bias voltage is 45 V, leading to the maximum available energy per cycle of *Model R* of 0.45 µJ, (3 *g*_peak_, 12 Hz), the maximum power is 13.2 μW (3 *g*_peak_, 50 Hz) and the maximum effectiveness is 54% (2 *g*, 10 Hz).

The output of *Model R* with varied bias voltage is converted to DC voltage through a diode bridge rectifier, and the average power is calculated according to the charging curves. The optimal operation point is found to be *V*_bias_ = 50 V, *V*_res_ = 12 V, corresponding to the energy conversion rate of 64.4 nJ/cycle (2 *g*_peak_, 10 Hz). The AC/DC converted energy of *Model R* is used as the power supply of an RFID tag for data transmission. The duration of each charging is 16 s (3 *g*_peak_, 11 Hz). With a 3-Hz hand shaking (2 *g*_peak_), the charging period is 2.2 min.

## Electronic supplementary material

Figure S5

Supplementary materials

Figure S1

Figure S2

Table S1

Figure S3

Figure S4

Table S2
