# Supplementary figures and images for: A power supply module for autonomous portable electronics: ultralow-frequency MEMS electrostatic kinetic energy harvester with a comb structure reducing air damping
Source: Microsyst Nanoeng. 2018 Sep 24;4:28. doi: 10.1038/s41378-018-0025-2 (PMC6220193; doi:10.1038/s41378-018-0025-2)

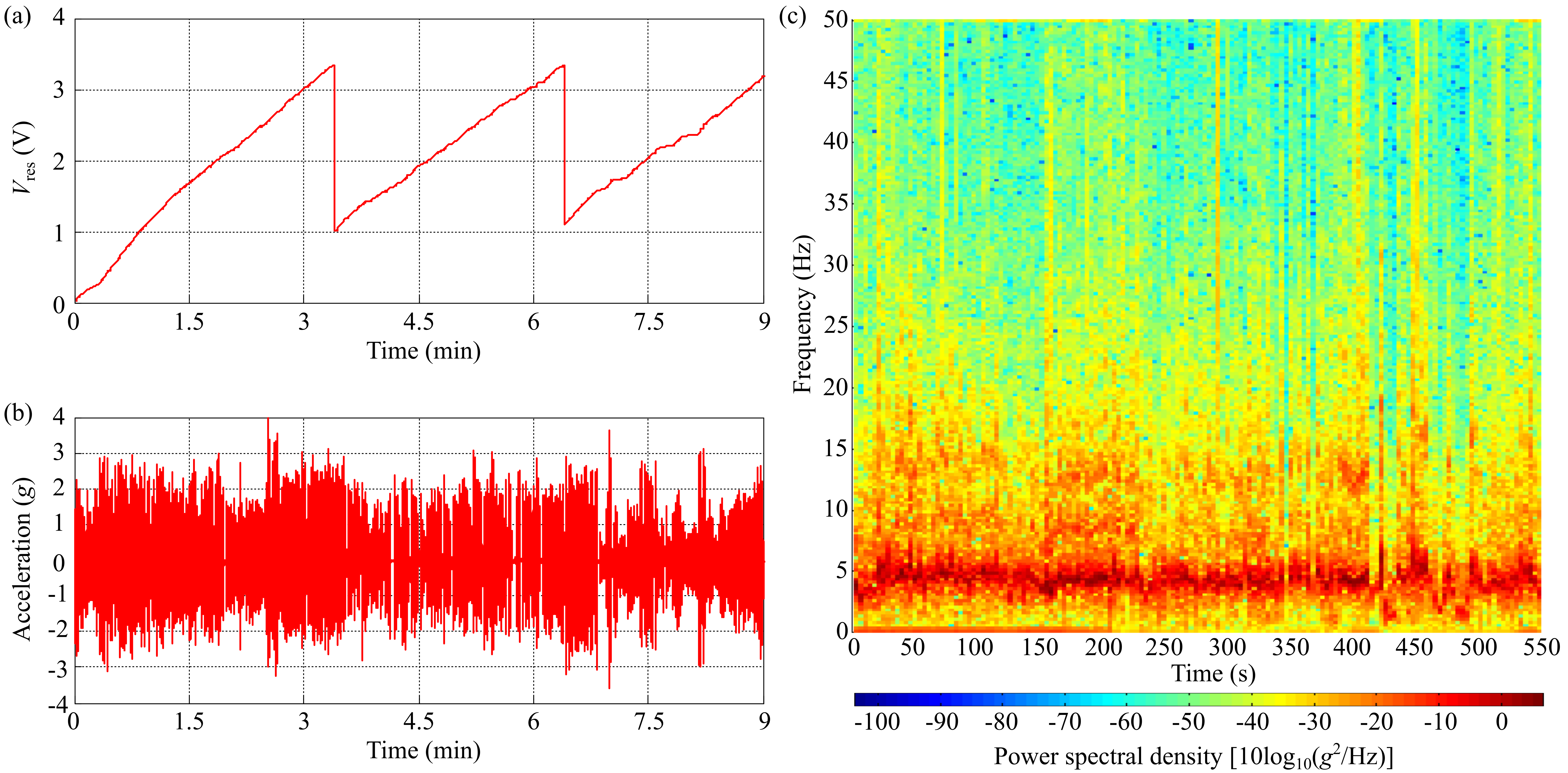

Supplement: Supplementary file 1 — Figure S5 [file 41378_2018_25_MOESM1_ESM.tif]

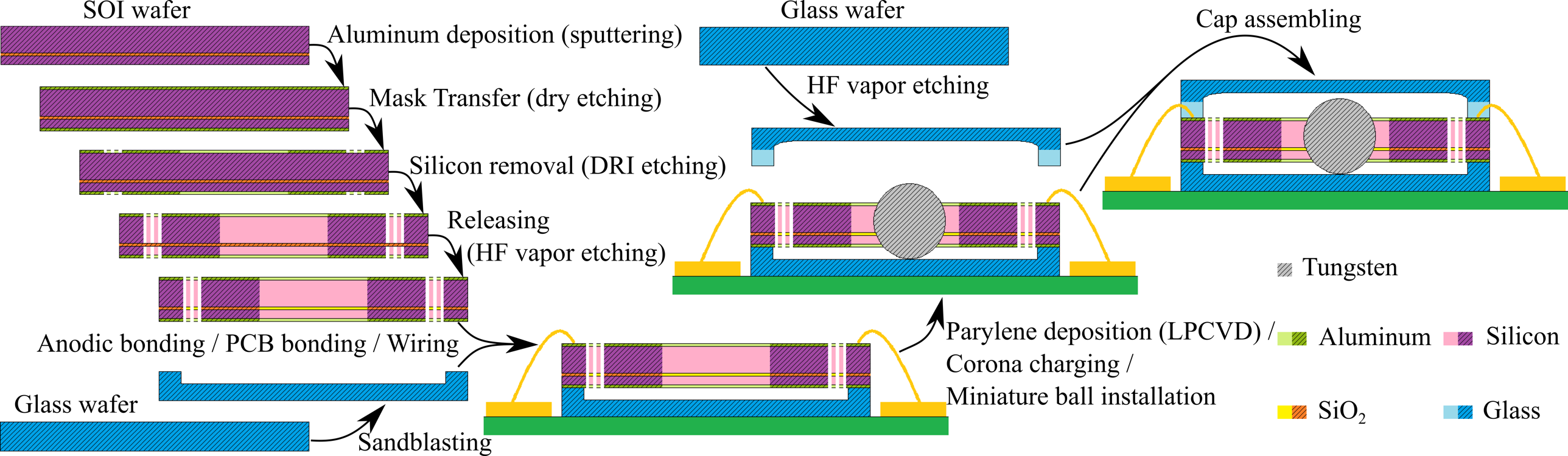

Supplement: Supplementary file 3 — Figure S1 [file 41378_2018_25_MOESM3_ESM.tif]

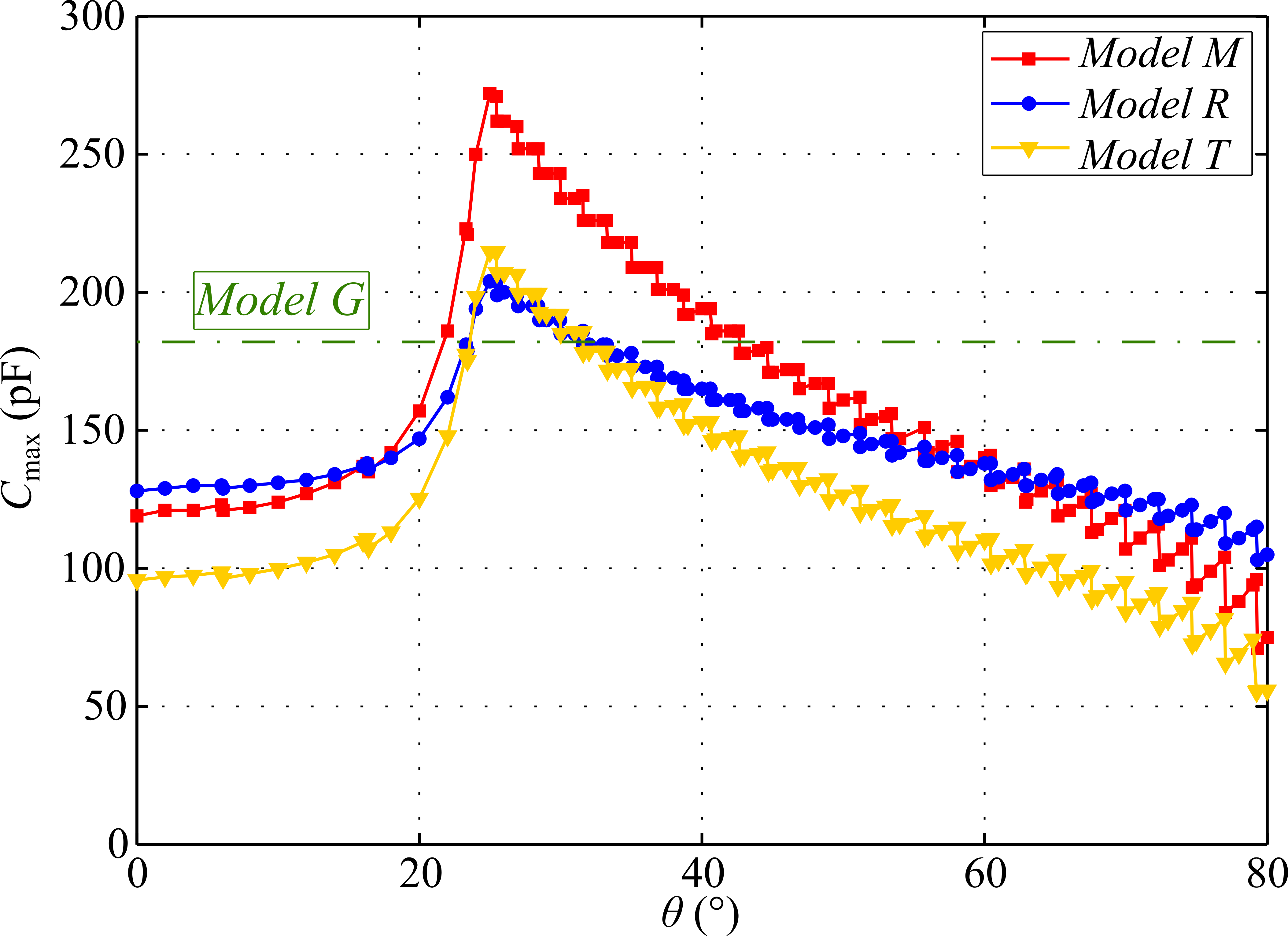

Supplement: Supplementary file 4 — Figure S2 [file 41378_2018_25_MOESM4_ESM.tif]

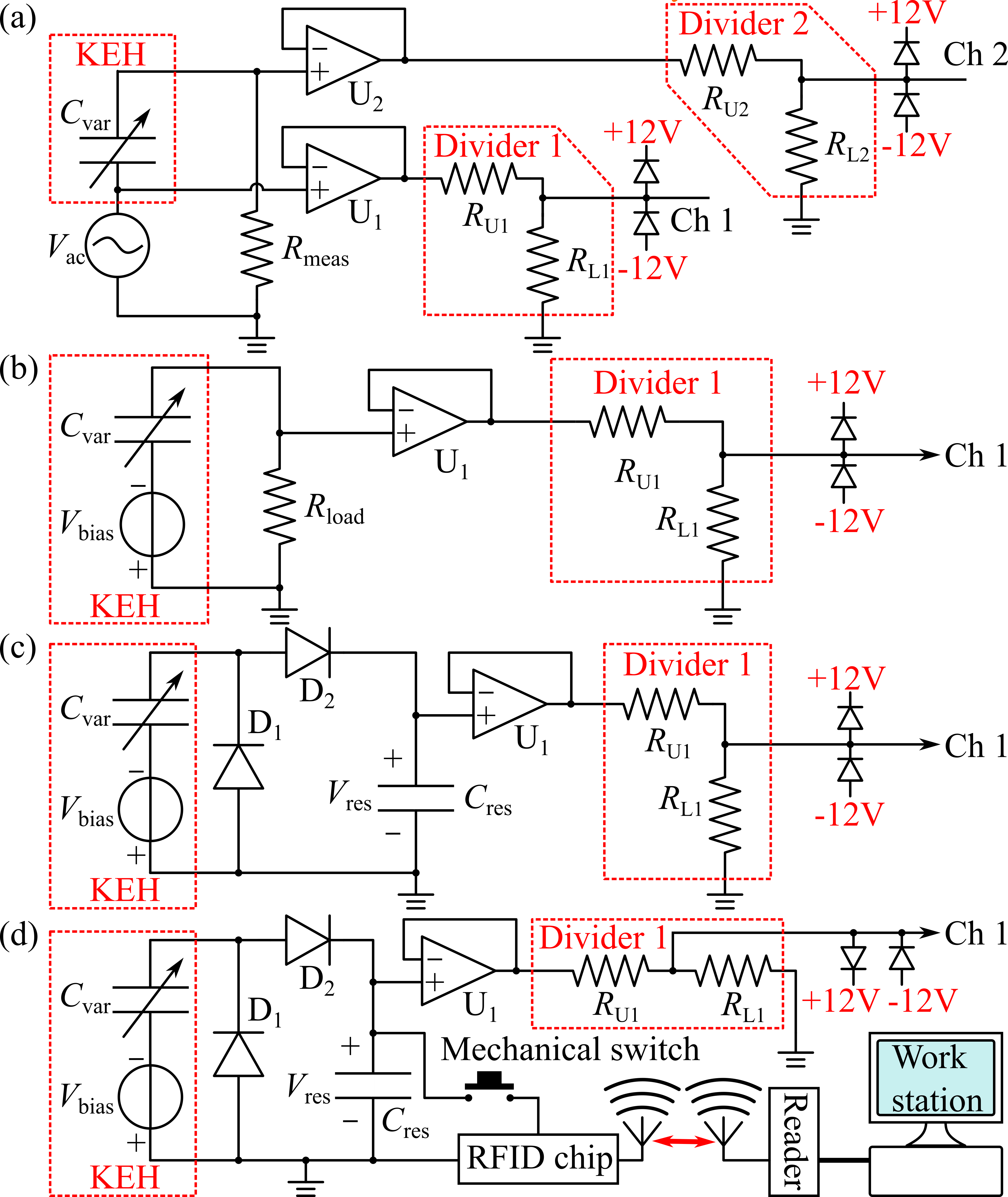

Supplement: Supplementary file 7 — Figure S4 [file 41378_2018_25_MOESM7_ESM.tif]
