# Supplementary material for: A power supply module for autonomous portable electronics: ultralow-frequency MEMS electrostatic kinetic energy harvester with a comb structure reducing air damping
Source: Microsyst Nanoeng. 2018 Sep 24;4:28. doi: 10.1038/s41378-018-0025-2 (PMC6220193; doi:10.1038/s41378-018-0025-2)
Supplement: Supplementary file 2 — Supplementary materials [file 41378_2018_25_MOESM2_ESM.pdf]

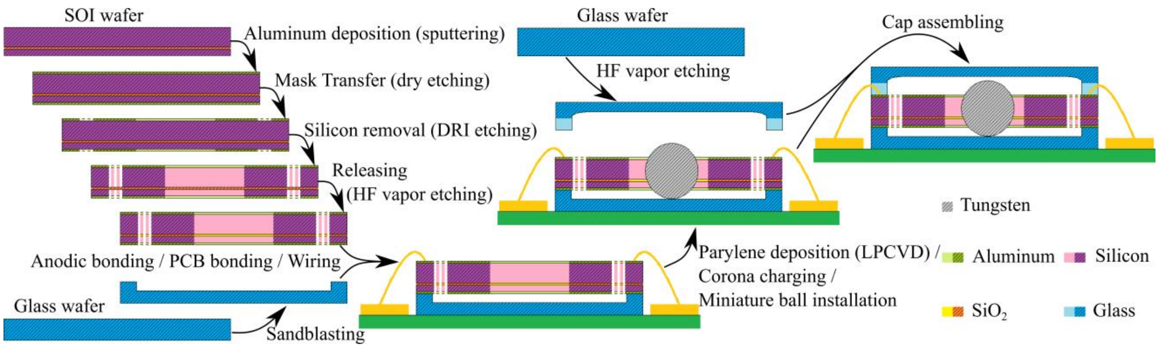

Figure S1 Fabrication process of the prototype

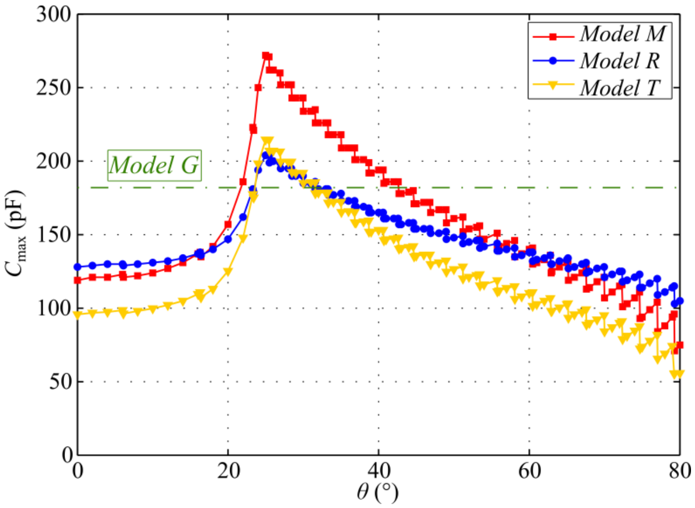

Figure S2 Parametric optimization: The influence of θ on C_{max} (corresponding to the displacement of 66 μm) of the 3 models with hierarchical combs (M, R & T).

Table S1 Design parameters of the prototypes (Models G, R, M, T)

Quantity	Symbol	Value
Thickness of handle layer	T_1	380 μm
Thickness of device layer	T_2	100 μm
Thickness of BOX layer	T_{ox}	2 μm
Finger length	L	2 mm
Finger width (defined in the mask layer)	W	20 μm
Number of fingers on the movable electrode	N_{f}	100
Initial gap between fingers on the planar sides (defined in the mask layer)	G_1	70 μm
Initial gap between fingers on the sides with teeth structure (defined in the mask layer)	G_2	140 μm
Tooth height	h_{tooth}	70 μm
Width of the tooth tip	W_{tooth}	30 μm
Tooth angle	θ	30°
Number of teeth on each side of a finger	N_{t}	14
Overlapping length of combs	L_{OL}	1.9 mm
Position of the stoppers	d_{st}	68 μm
Mass of the movable electrode	m	77.5 mg (Model G)<br>79.4 mg (Models R & M)<br>80.4 mg (Model T)
Mass of the miniature ball	m_{b}	32 mg
Total stiffness of the linear springs	k	40.5 N/m
Total stiffness of the elastic stoppers	k_{st}	1.02×10^4 N/m
Cavity length	L_{cav}	3 mm
Radius of the miniature ball	r	0.8 mm

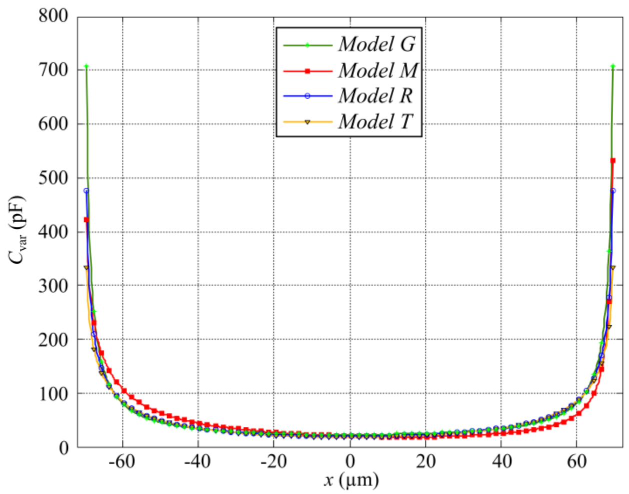

Figure S3 Capacitance variation of the 4 models with θ=30° in relation with the mass displacement (predicted by analytical model). The capacitances close to C_{min} are less accurate than the values close to C_{max}, because the influence of inter-layer structure is ineligible when the gap between electrodes is large, but not taken into account.

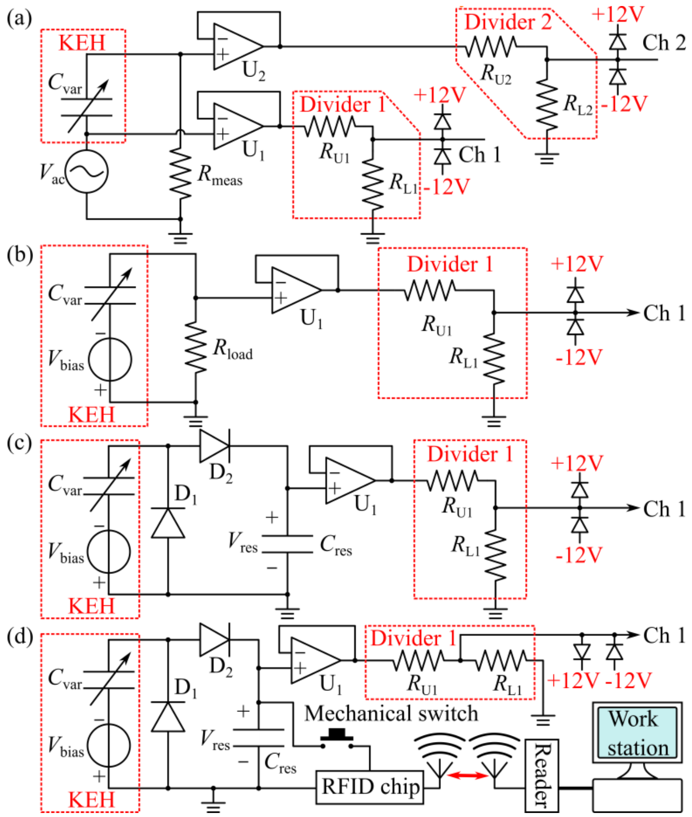

Figure S4 Experimental setup: (a) Schematics of the circuits for experiments including: dynamic capacitance measurement; (b) AC power measurement with resistive load; (c) AC/DC power measurement with diode bridge rectifier; (d) data transmission experiment.

Table S2 Capacitance variation (in pF) and ratio of the 4 models measured in air / vacuum at 2 g_{peak} at optimal frequency.

	In air			In vacuum		
	$C_{\max}$	$C_{\min}$	η	$C_{\max}$	$C_{\min}$	η
Model G	130	25	5.2	440	25	17.6
Model T	200	25	8	200	25	8
Model R	270	25	10.8	290	25	11.6
Model M	120	25	4.8	230	25	9.2

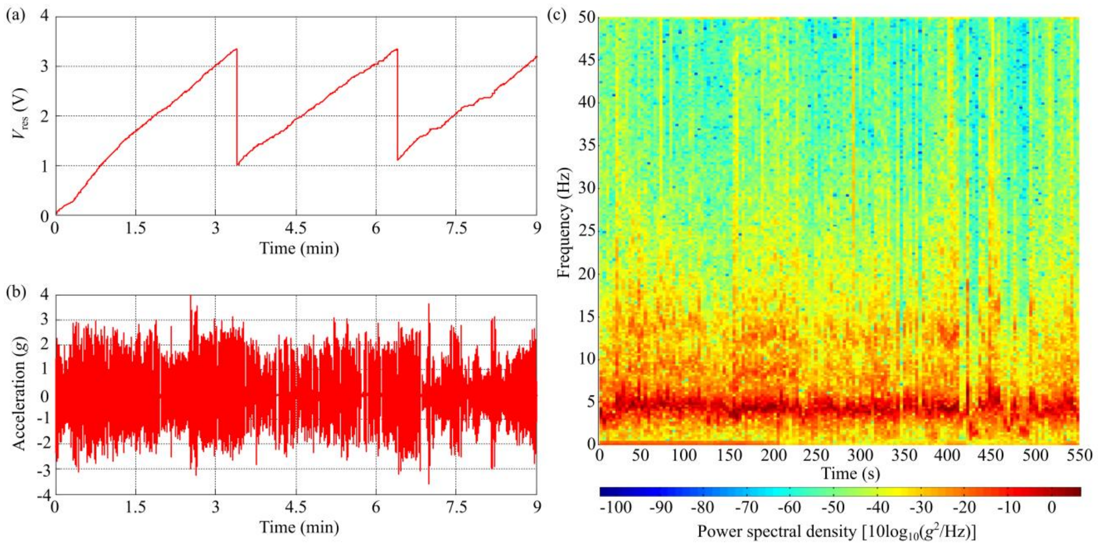

Figure S5 Data transmission experiment when the KEH (*Model R*) is biased at 50 V and excited by a random hand-shaking motion: (a) V_{res} evolution (energy conservation / release) during the experiment (b) acceleration recorded by an accelerometer attached to the KEH (the root mean square of the signal is about 0.91g) (c) power spectral density evolution of the hand-shaking acceleration.
