# Supplementary material for: A power supply module for autonomous portable electronics: ultralow-frequency MEMS electrostatic kinetic energy harvester with a comb structure reducing air damping
Source: Microsyst Nanoeng. 2018 Sep 24;4:28. doi: 10.1038/s41378-018-0025-2 (PMC6220193; doi:10.1038/s41378-018-0025-2)
Supplement: Supplementary file 5 — Table S1 [file 41378_2018_25_MOESM5_ESM.pdf]

Table S1 Design parameters of the prototypes (Models G, R, M, T)

Quantity	Symbol	Value
Thickness of handle layer	T_1	380 µm
Thickness of device layer	T_2	100 µm
Thickness of BOX layer	T_{ox}	2 µm
Finger length	L	2 mm
Finger width (defined in the mask layer)	W	20 µm
Number of fingers on the movable electrode	N_{f}	100
Initial gap between fingers on the planar sides (defined in the mask layer)	G_1	70 µm
Initial gap between fingers on the sides with teeth structure (defined in the mask layer)	G_2	140 µm
Tooth height	h_{tooth}	70 µm
Width of the tooth tip	W_{tooth}	30 µm
Tooth angle	θ	30°
Number of teeth on each side of a finger	N_{t}	14
Overlapping length of combs	L_{OL}	1.9 mm
Position of the stoppers	d_{st}	68 µm
Mass of the movable electrode	m	77.5 mg (Model G)<br>79.4 mg (Models R & M)<br>80.4 mg (Model T)
Mass of the miniature ball	m_{b}	32 mg
Total stiffness of the linear springs	k	40.5 N/m
Total stiffness of the elastic stoppers	k_{st}	1.02×10^4 N/m
Cavity length	L_{cav}	3 mm
Radius of the miniature ball	r	0.8 mm
