# Supplementary material for: A power supply module for autonomous portable electronics: ultralow-frequency MEMS electrostatic kinetic energy harvester with a comb structure reducing air damping
Source: Microsyst Nanoeng. 2018 Sep 24;4:28. doi: 10.1038/s41378-018-0025-2 (PMC6220193; doi:10.1038/s41378-018-0025-2)
Supplement: Supplementary file 8 — Table S2 [file 41378_2018_25_MOESM8_ESM.pdf]

Table S2 Capacitance variation (in pF) and ratio of the 4 models measured in air / vacuum at 2 g_{peak} at optimal frequency.

	In air			In vacuum		
	$C_{\max}$	$C_{\min}$	η	$C_{\max}$	$C_{\min}$	η
Model G	130	25	5.2	440	25	17.6
Model T	200	25	8	200	25	8
Model R	270	25	10.8	290	25	11.6
Model M	120	25	4.8	230	25	9.2
